# Multi-host Model-Based Identification of *Armillifer agkistrodontis* (Pentastomida), a New Zoonotic Parasite from China

**DOI:** 10.1371/journal.pntd.0000647

**Published:** 2010-04-06

**Authors:** Shao-Hong Chen, Qin Liu, Yong-Nian Zhang, Jia-Xu Chen, Hao Li, Ying Chen, Peter Steinmann, Xiao-Nong Zhou

**Affiliations:** 1 National Institute of Parasitic Disease, Chinese Center for Disease Control and Prevention, WHO Collaborating Center of Malaria, Schistosomiasis and Filariasis, Shanghai, People's Republic of China; 2 Department of Epidemiology and Public Health, Swiss Tropical and Public Health Institute, Basel, Switzerland; George Washington University, United States of America

## Abstract

**Background:**

Pentastomiasis is a rare parasitic infection of humans. Pentastomids are dioecious obligate parasites requiring multiple hosts to complete their lifecycle. Despite their worm-like appearance, they are commonly placed into a separate sub-class of the subphylum Crustacea, phylum Arthropoda. However, their systematic position is not uncontested and historically, they have been considered as a separate phylum.

**Methodology/Principal Findings:**

An appraisal of *Armillifer agkistrodontis* was performed in terms of morphology and genetic identification after its lifecycle had been established in a multi-host model, i.e., mice and rats as intermediate hosts, and snakes (*Agkistrodon acutus* and *Python molurus*) as definitive hosts. Different stages of the parasite, including eggs, larvae and adults, were isolated and examined morphologically using light and electron microscopes. Phylogenetic and cluster analysis were also undertaken, focusing on the 18S rRNA and the Cox1 gene. The time for lifecycle completion was about 14 months, including 4 months for the development of eggs to infectious larvae in the intermediate host and 10 months for infectious larvae to mature in the final host. The main morphological difference between *A. armillatus* and *Linguatula serrata* is the number of abdominal annuli. Based on the 18S rRNA sequence, the shortest hereditary distance was found between *A. agkistrodontis* and *Raillietiella* spp. The highest degree of homology in the Cox 1 nucleic acid sequences and predicted amino acid sequences was found between *A. agkistrodontis* and *A. armillatus*.

**Conclusion:**

This is the first time that a multi-host model of the entire lifecycle of *A. agkistrodontis* has been established. Morphologic and genetic analyses supported the notion that pentastomids should be placed into the phylum Arthropoda.

## Author Summary

Little information is currently available on the lifecycle and morphology of pentastomids, a new zoonotic parasite in China. The lifecycle of *Armillifer agkistrodontis* was established in multiple hosts, i.e., an intermediate host and a definitive host, and the parasite examined in terms of morphology and genetic relationship with other species. The time required for the completion of an entire lifecycle was about 14 months. The main morphological difference between *A. armillatus* and *L. serrata* is the number of abdominal annuli. The genetic data supported the notion that pentastomids belong to the phylum Arthropoda. Based on the 18S rRNA sequence, the shortest hereditary distance was found between *A. agkistrodontis* and *Raillietiella* spp. The highest similarity in the Cox 1 nucleic acid sequences was found between *A. agkistrodontis* and *A. armillatus*. The established multi-host model provides a possible approach to confirm suspected infections and offers an opportunity to further study this parasite.

## Introduction

Human pentastomiasis is a food- or water-borne parasitic zoonosis caused by pentastomids or “tongue worms” [1]. Pentastomida are dioecious obligate parasites commonly placed into a separate sub-class of the subphylum Crustacea, phylum Arthropoda, despite their worm-like appearance. However, their systematic position is not uncontested and historically, they have been considered as a separate phylum. Adult pentastomids usually reside in the respiratory tract of their reptile, bird or mammal end hosts while larvae live in the internal organs of vertebrate or arthropod intermediate hosts [2], [3]. Pentastomids belonging to the two genera *Linguatula* and *Armillifer* are implicated in the majority of all human infections [4], [5]; the two species *L. serrata* and *A. armillatus* account for more than 90% of all reported pentastomiasis cases [6], [7]. The four pathogenic *Armillifer* species are *A. armillatus* in Africa and the Arabian Peninsula, *A. agkistrodontis* in China, *A. grandis* in Africa and *A. moniliformis* in Southeast Asia. The lifecycle of *Armillifer* spp. is believed to alternate between a snake - mouse cycle and one involving snakes and other mammals [8]. Snakes are the main source of infection for humans and local cultural idiosyncrasies are the main drivers for infection [8]. Cases involving other members of the genus than *A. armillatus* are rare but a number of human infections with *A. agkistrodontis* have been reported in China over recent years [9]–[13]. Drinking water contaminated with infectious eggs and the consumption of uncooked snake blood and gallbladders were identified as the likely sources of these infections [11], [13]. However, almost no data is available about the biology and natural history of *A. agkistrodontis*, including its lifecycle and morphological observations [14], [15].

The aim of the present study was i) to unambiguously identify the recently found parasites as *A. agkistrodontis* after the establishment of the entire lifecycle in multiple hosts in the laboratory; ii) to describe the morphological and biological characteristics of different developmental stages of *A. agkistrodontis*; and iii) to perform phylogenetic comparisons between this species and other pentastomids in order to confirming its systematic position.

## Materials and Methods

### Parasites and lifecycle establishment

Two *Agkistrodon acutus* snakes trapped in Fuyang county, Zhejiang province, China, were confirmed to be infected with suspected *A. agkistrodontis* after dissection. An adult gravid *A. agkistrodontis* specimen was isolated using standard procedures [16], [17], and mature eggs were recovered after culture for 1–2 h in an incubator at 26±0.5°C and 80% relative humidity. Using these eggs, infection of four different potential intermediate hosts was attempted. First, a mouse model was established in 100 female Kunming mice aged 6∼8 weeks (body weight: 20±5 g). Each mouse was infected with a total of 40 mature *A. agkistrodontis* eggs via a plastic tube reaching the stomach. Second, 8 female SD rats aged 6∼8 weeks and weighing 100±5 g were infected with 80 eggs each using a similar procedure. Third, five New Zealand purebred big white rabbits with a body weight of 1.5∼2 kg were infected with 600 eggs each, again via intubation. Last, 5 female dogs (body weight: 3.5∼5 kg) were fed 600 eggs each with their regular food.

Three infected mice were dissected each week between week 1 and 22, and the presence of larvae as well as their distribution and the recovery-ratio were determined. Once infectious larvae had been found in mice, one rat, one rabbit and one dog were dissected every two weeks in order to also establish the developmental speed, distribution and larvae recovery-ratio in these host animals.

A total of seven snakes, 4 *A. acutus* (toxic) and 3 *Python molurus*, were raised in the Veterinary Hospital of Shanghai Zoo, and fed with 4-month old mice confirmed to be infected with *A. agkistrodontis* by ELISA and isolation of parasite larvae. The ELISA included the following steps. First, Immulon 2HB plates were coated overnight with purified soluble antigen at a concentration of 1 µg/well in 0.05M carbonate buffer (pH 9.6). The antigen had been prepared using *A. agkistrodontis* larvae. Wells were blocked overnight with 5% skimmed milk in phosphate-buffered saline containing 0.05% Tween 20. Sera were diluted 1∶320 in PBST containing 5% skimmed milk and incubated for 1h at 37°C. Then, wells were washed with PBST and goat anti-mouse IgG (Fc fragment-specific) HRP conjugate was added at a 1∶4000 dilution in PBST and incubated for 1h at 37°C. Last, plates were washed and the signal measured at 490nm after addition of OPD and 2M H_2_SO_4_ to end the reaction. PBS and sera from uninfected mice were used as negative controls. The mean OD value of 80 normal mice sera was used as the negative reference value. A ratio of infected mouse sera/negative reference ≥2.0 was set as the cut-off value. Regular fecal examination was performed on all snakes from day 60 post-infection onwards to determine the time until immature and mature eggs were shed. Once fully developed eggs had been recovered, the snakes were dissected and their worm burden determined.

### Microscopic examination

The morphological examination of *A. agkistrodontis* eggs, larvae and adults was performed using an Olympus BX51 microscope and an Olympus BX12 stereo microscope, as appropriate. The shape and structure of both larvae and adult parasites were studied, and the mean size of mature and immature eggs was determined by measuring 100 mature eggs and 100 immature eggs.

Representative *A. agkistrodontis* eggs, larvae and adults were embedded in epoxy resin after appropriate preparation steps, i.e., cleaning, fixing, drying, and gold coating [18]. The embedded samples were sliced using a LKB-E super slicer, double stained, and examined under a Philips CM210 transmission electron microscope. Further samples were studied using a S-520 scanning electron microscope (Hitachi) after standard preparations [19], [20].

### Phylogenetic analysis

Genomic DNA was extracted from adult *A. agkistrodontis* according to a method described by Liu and colleagues [21]. Respective primers were designed for the 18S rRNA and Cox1 gene according to an *A. armillatus* mitochondrial sequence (GenBank Assession no. AY456186), and obtained from Shanghai Bio-Engineering Technical Service Limited Company, Shanghai, China. The primer sequences were as follows: 18S rRNA-F: (5′-AAC CTG GTT GAT CCT GCC AGT AG -3′), 18S rRNA-R: (5′-GAT CCT TCT GCA GGT TCA CCT AC-3′); Cox1-F: (5′-CTG CGA CAA TGA CTA TTT TCA AC), Cox1-R: (5′-ATA TGG GAA GTT CTG AGT AGG-3′). The polymerase chain reaction (PCR) was performed on a C_1000_™ Thermal cycler (Bio-RAD) using 2.0µl genomic *A. agkistrodontis* DNA as template. The reaction conditions were: 96°C for 3 min followed by 33 cycles with 94°C for 30 sec, 58°C for 30 sec, 72°C for 2 min. The last step was at 72°C for 10 min. The product was visualized on an agarose gel using ethidium bromide staining. The target DNA fragments were inserted into pGEM-T easy vectors (Promega) after recycling and purifying using a commercial kit (Unit-10 reception kit, Sangon, Shanghai, China). The plasmids were transformed into *E. coli* DH5α, and small amounts of plasmids were recovered for identification of the insert after enzymatic digestion. Positive clones were sequenced from their 5′ and 3′ ends, respectively (Shanghai Dingan Biotechnology Ltd. Co.).

The sequence was then submitted to the NCBI GenBank, and its homology with regard to nucleotide and amino acid sequence compared to available sequences using the BLAST procedure [21], [22]. The 18S rRNA sequence of *A. agkistrodontis* was matched with the corresponding sequences of representative species using Clustal×1.8, and the Clustal W output file was inverted into a meg-formatted document using MEGA4.1. The relationship analysis and phylogenetic tree development were performed by Neighbor-Joining (N-J) and 1000 Bootstrap analyses using as reference the 18S rRNA outgroup sequence of *Speleonectes tulumensis* (L81936) [5].

### Ethical considerations

Both the Scientific Committee and the Ethical Committee of the National Institute of Parasitic Diseases, China CDC reviewed and approved the study proposal. All study procedures were conducted in adherence to institutional guidelines for animal husbandry.

## Results

### Parasite lifecycle

*A. agkistrodontis* infections were successfully established in the mouse and rat models but no larvae could be recovered from rabbits and dogs previously fed *A. agkistrodontis* eggs. Mean larval recovery rates were 79.2% in mice and 51.2% in rats (Table 1).

**Table 1:** Infective dose and outcome of experimental *A. agkistrodontis* infections in four potential intermediate hosts.

Immediate host	No. of eggs for infection	Mean no. of larvae recovered	Larval recovery rate (%)	Time until first larvae appeared (weeks)
Mouse	40	31.7	79.2	11
Rat	80	41	51.2	11
Rabbit	600	0	0	n.a.
Dog	600	0	0	n.a.

n.a.: not applicable.

No changes were observed in the viscera of the mice dissected between week 1 and 5 post-infection. In week 6, the bleeding point and 1∼2 nodules appeared in the liver and spleen. In subsequent weeks, the size of the nodules significantly increased. The first larvae were observed 11 weeks post-infection, and larvae had reached the infectious stage after 16 weeks. Twenty-two weeks post-infection, larvae were found in the mesentery (66.0% of the total number of larvae), liver (15.0%), spleen (13.9%), thoracic cavity (4.4%) and in the lung (0.9%), owing to a recovery rate of 79.2%. In rats, the larval recovery rate was 51.2%; 70.0% of the larvae resided in the mesentery 11 weeks post-infection and another 18.6% in the spleen, 4.0% in the liver and 3.2% in each the kidney and the thoracic cavity. No larvae were found in the rats' lungs.

**Figure 1:**
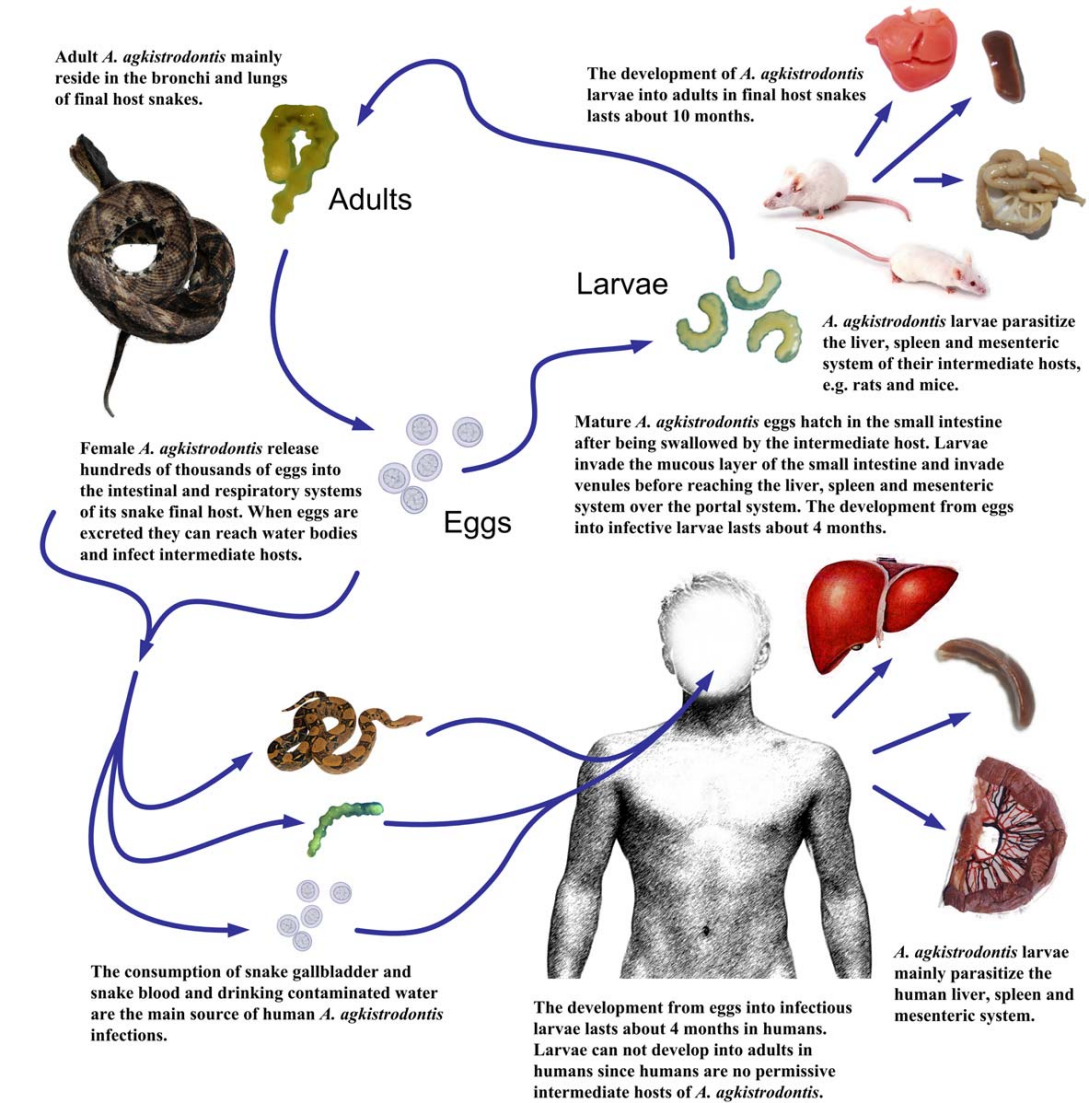
Lifecycle of *A. agkistrodontis*.

The established *A. agkistrodontis* lifecycle under laboratory conditions is depicted in Figure 1. Mature eggs were first observed in the feces of the final host snakes, i.e., *A. acutus* and *P. molurus*, after a prepatent period of 10 months, suggesting a minimum period of 14 months required to complete the lifecycle in the hosts studied here and under laboratory conditions. Adult parasites mainly resided in the abdominal cavity 5–6 months post-infection but the focus of their localization shifted to the lungs about 9–10 months after infection (Table 2).

**Table 2:** Change in location of *A. agkistrodontis* larvae in definitive host snakes over time.

Hosts	Months post-infection	Faecal examination	No. of larvae in lungs	No. of larvae in bronchia	No. of larvae in abdominal cavity	Total no. of larvae
*A. acutus* no. 1	4.5	negative	0	0	60	60
*P. molurus* no. 2	5	negative	0	0	26	26
*P. molurus* no. 3	5	negative	0	0	5	5
*P. molurus* no. 4	6	negative	0	2	22	24
*A. acutus* no. 5	9	negative	8	0	0	8
*A. acutus* no. 6	10	positive	8	0	0	8
*A. acutus* no. 7	10	positive	7	0	0	7

### Morphological observations

**Figure 2:**
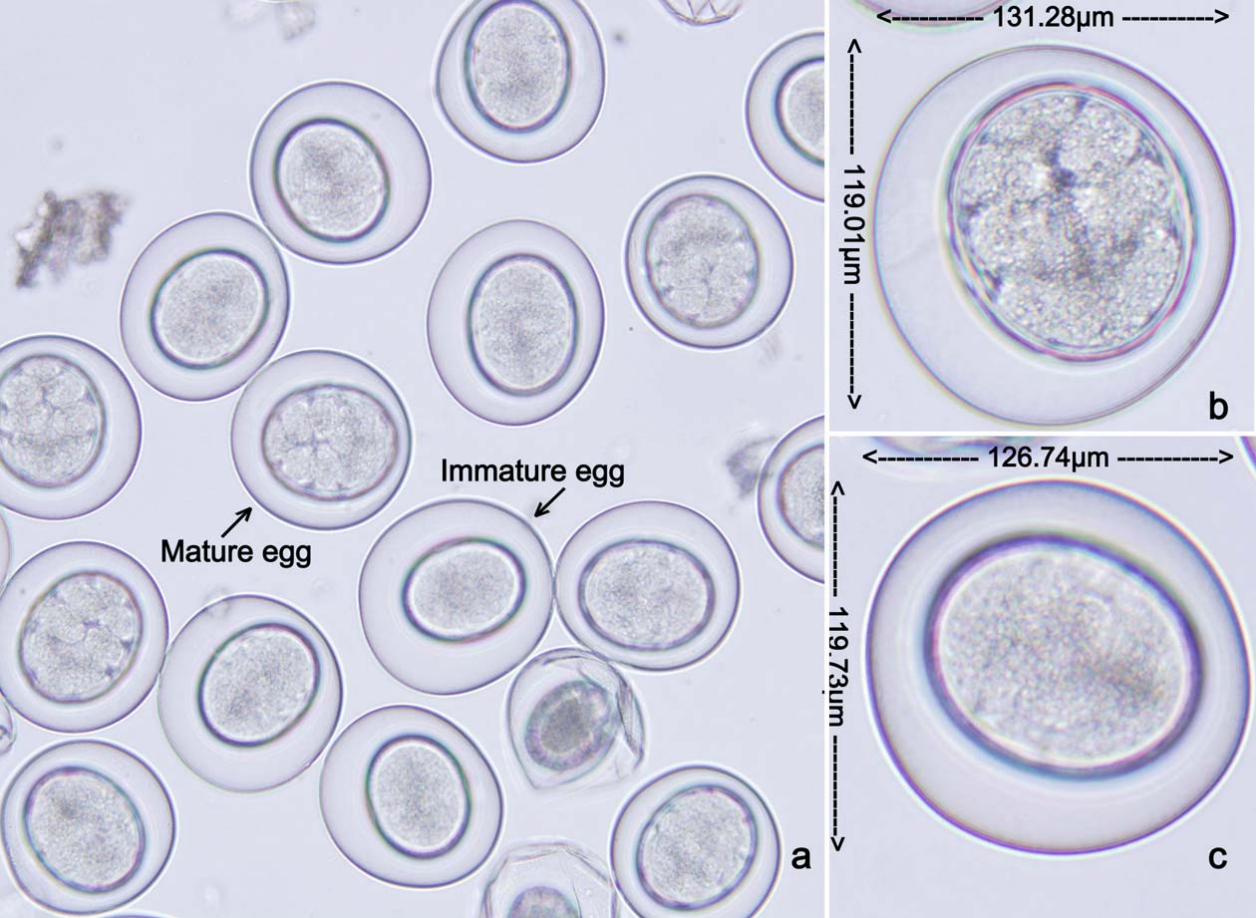
*A. agkistrodontis* eggs as seen under the microscope. a: Mature and immature eggs isolated from uterus; b: mature egg isolated from uterus; c: immature egg isolated from uterus.

Both mature and immature *A. agkistrodontis* eggs had an oval shape and two layers could be distinguished. The outer layer was transparent with a whitish colour while the inner layer appeared yellow. In mature eggs, a typical arthropod larva could be observed while immature eggs contained yolk cells (Figure 2). The mean overall length and width of mature and immature eggs was 131.28 µm×119.01 µm and 126.74 µm×119.73 µm respectively (Table 3).

**Table 3:** Average size of immature and mature *A. agkistrodontis* eggs recovered from definitive host snakes.

Egg	Length of outer layer (µm)	Width of outer layer (µm)	Length of inner layer (µm)	Width of inner layer (µm)
Mature egg	131.28±8.37	119.01±8.97	86.36±8.46	67.73±8.38
Immature egg	126.74±7.71	119.73±7.64	86.36±6.09	54.68±5.84

**Figure 3:**
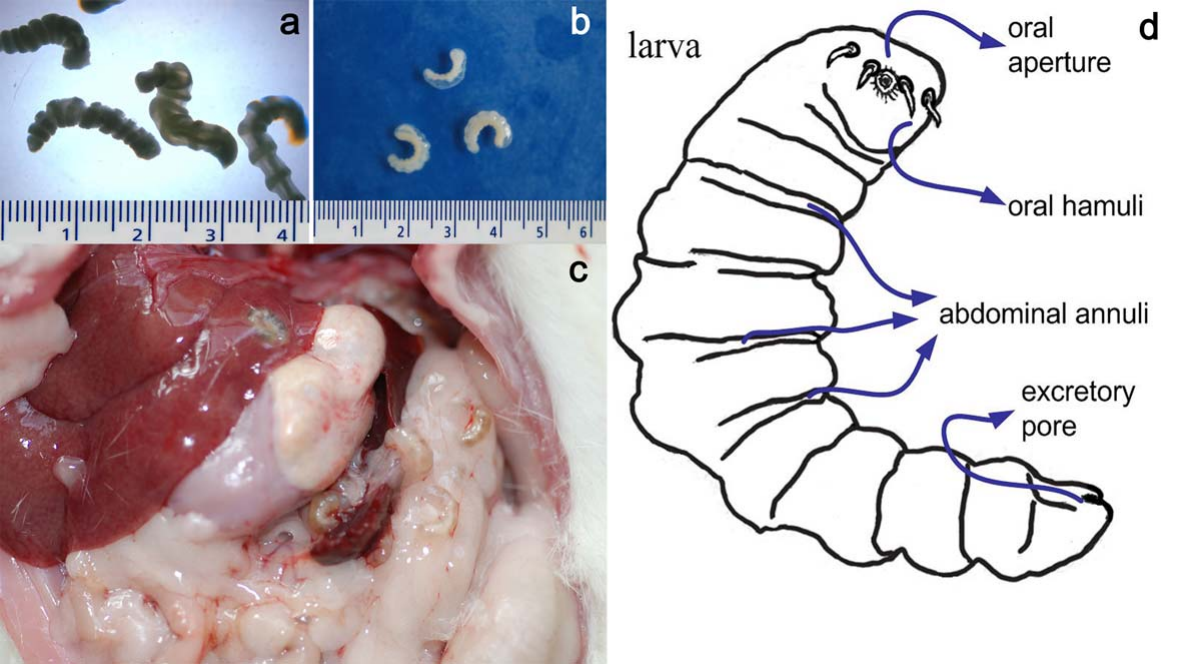
Morphology of *A. agkistrodontis* larvae. a: Living larvae; b: dead larvae; c: larvae in the liver and spleen of a mouse; d: schematic representation of a larva showing different structures on the body surface.

The sequential observation of larvae recovered from mice showed that after 22 weeks, *A. agkistrodontis* larvae had developed a cylinder-shaped body widened at the forehead and narrowed towards the posterior end. Larvae appeared transparent when alive (Figure 3a) but turned to a creamy color after they had died (Figure 3b). Larvae living in the mouse were C-shaped and covered by a thin pouch with a fibrous structure (Figure 3c). The mean length of larvae was 16.75±1.23 mm (range: 15–19 mm) and their mean width was 2.82±0.24 mm; they possessed 7–9 abdominal annuli which gradually increased in length from the head towards the tail. Wave-like folds were also observed on the body surface between the abdominal annuli. The oral aperture was located in the forehead, and hamuli as well as the excretory pore were located on the abdominal side of the rear body. A pair of curved hamuli was observed on each side of the oral aperture. Figure 3d is a schematic representation of a larva.

**Figure 4:**
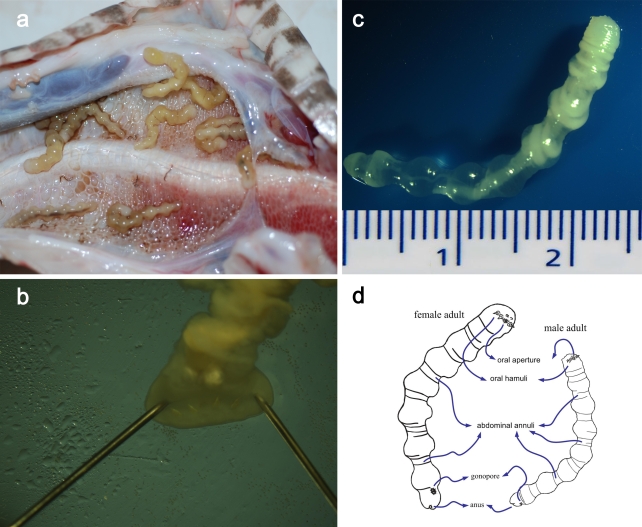
Morphology of adult *A. agkistrodontis*. a: Adults in the lung and thorax of a snake; b: oral cavity of an adult; c: life adult; d: schematic representation of an adult male and an adult female showing different structures on the body surface.

Mature parasites (Figure 4a) were translucent and females measured 42.71±7.11 mm (range: 33–48 mm) ×5.27±0.73 mm (range: 4.1–6.3 mm). The oval mouth was located on the abdominal side of the cephalothorax. Two horizontal pairs of hamuli were located on each side of the mouth. A pair of mastoid processes could be observed anterior to the mouth, and a pair of golden ovum-shaped pellets was observed behind the mouth and extending into the esophagus (Figure 4b). The chest cavity was opaque and yellow-whitish; the bilateral body transparent. It appeared to be filled with eggs. Both females and males had 7–9 abdominal annuli with a cutin structure in bangle shape on the abdominal side. The body wall between the abdominal annuli was transparent and thin (Figure 4c). The anus was located terminally, behind the gonopore. Adult males measured 23.96±0.72 mm (range: 23–25 mm) ×4.0±0.58 mm (range: 3.2–4.8 mm) with a general anatomical structure similar to that observed in females. The male gonopore was located in the abdominal median line in the terminal section of the body. A schematic representation of male and female adult *A. agkistrodontis* is shown in Figure 4d.

### Micro-morphological characteristics

**Figure 5:**
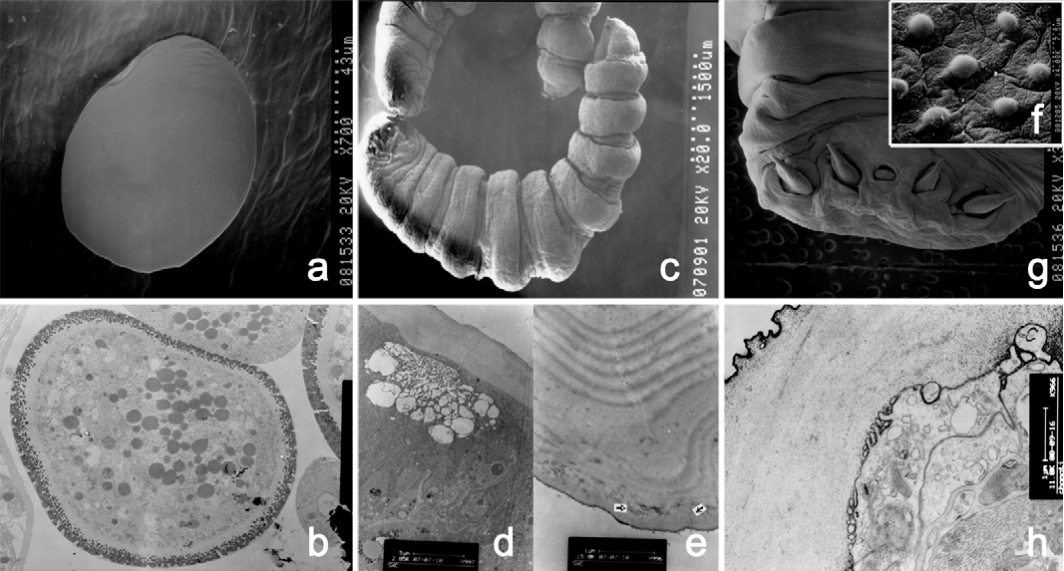
Morphological details of *A. agkistrodontis* seen under an electron microscope. a: Egg; scanning electron microscope (SEM); b: egg with three layers; transmission electron microscope (TEM); c: larva; SEM; d, e: larva with seven cortical layers; TEM; f, g: adult with a pair of curved hamuli on both sides of the mouth (f) and small masoid process-like spines on the body surface (g); SEM; h: adult with three cortical layers; TEM.

*A. agkistrodontis* eggs appeared oval in shape and covered by three layers, i.e., an outer membrane, a transparent layer and a medullary layer, when studied under the transmission electron microscope (Figure 5a, 5b). A pair of curved hamuli was observed on both sides of the oral aperture of larvae. Several sucker-like sensory organs of different size were distributed around the mouth, outside the hooks and on the abdominal annuli. Waved folds were noted on the body surface between the abdominal annuli. Granulation-like excretory pores appeared on the abdominal side of the front part (Figure 5c). Seven cortical layers could be distinguished under the transmission electron microscope, namely the outer cortex, lucidity layer, inner cortex, homogenous layer, medulla, striated layer and base plate (Figure 5d, 5e). The morphological characterization of *A. agkistrodontis* larvae appeared different from those of *L. serrata* and *P. taiwana*. The cephalic part of *A. agkistrodontis* is columniform while that of *L. serrata* and *P. taiwana* is conic or round respectively. The oral aperture of *A. agkistrodontis* is oval and located on the abdominal side in the center of the forehead while those of *L. serrata* and *P. taiwana* are square or annular respectively. The number of abdominal annuli of *A. agkistrodontis* is 7∼9 while *L. serrata* and *P. taiwana* have about 80 and 10∼11 respectively. These features can thus be employed to differentiate the three species (Qiu MH, personal communication).

Small mastoid process-like spines covered the entire body surface of adult *A. agkistrodontis* (Figure 5f), and a pair of curved hamuli was located on each side of the mouth (Figure 5g). Raised oval sensory organs resembling pores were scattered along both sides of the front and the posterior part of the body, and the anal pore was located terminally. Three cortical layers were noted under the transmission electron microscope, namely an outer cortex, an inner cortex and the base plate (Figure 5h).

### Phylogenetic analysis

**Figure 6:**
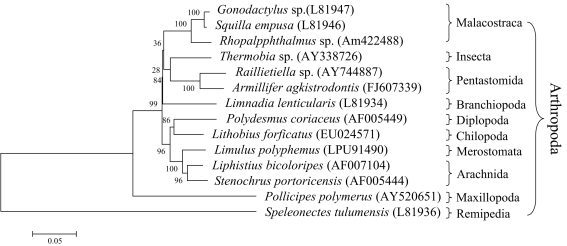
Genetic relationship between *A. agkistrodontis* and other species of the phylum Arthropoda, depicted as phylogenetic tree based on the Neighbor-Joining approach.

The nucleotide sequences of the *A. agkistrodontis* 18S rRNA comprised 1835bp (GenBank Accession No. FJ607339). The sequence was most similar to that of *Raillietiella* spp. (GenBank Accession No. AY744887) with an agreement of 93.2%. The phylogenetic tree was established based on the full 18S rRNA sequence and suggested that *A. agkistrodontis* is most closely related to *Raillietiella* spp. (Pentastomida) but also has a close genetic relationship with *Thermobia* spp. (Insecta; GenBank Accession No. AY338726) (Figure 6).

The nucleotide sequences of the *A. agkistrodontis* Cox1 gene measured 1525bp (GenBank Accession No. FJ607340) and encoded a 508-amino acid peptide. Its nucleotide sequence was most similar to that of *A. armillatus* (GenBank Accession No. AY456186; similarity 85.3%). The predicted amino acid sequence of the *A. agkistrodontis* Cox1 gene-encoded peptide had an agreement of 95.9% with the respective sequence from *A. armillatus* (GenBank Accession No. YP025989) as calculated by BLASTx.

The cladogram linking the 18S rRNA of *A. agkistrodontis* with the homologue sequences of representative species from 7 classes retrieved from GenBank, i.e., Malacostraca, Insecta, Pentastomida, Branchiopoda, Diplopoda, Chilopoda and Merostomata, showed that the genetic distance between *A. agkistrodontis* and *Raillietiella* spp. was shorter than that to any other species (Figure 6).

## Discussion

Little data regarding the natural history, morphology and molecular biology of pentastomids including *A. agkistrodontis* have yet been reported besides some information on *Porocephalus crotali*, the lifecycle of which had been successfully established in a laboratory setting [16], [23], [24]. To our knowledge, this is the first time that the complete lifecycle of *A. agkistrodontis* has been established under laboratory condition, providing a platform to further study this parasite. Four important observations have been made during the present study. Firstly, in addition to humans also mice and rats can be infected with *A. agkistrodontis* and thus can serve as intermediate hosts. Secondly, the most obvious morphological difference between *A. agkistrodontis* and more common pentastomid parasites, i.e., *A. armillatus* and *L. serrata*, were the number of abdominal annuli, numbering 7–9 in *A. agkistrodontis* but 14–22 and 72–108 in *A. armillatus* and *L. serrata* respectively. Thirdly, a visceral larva migrans was observed in intermediate hosts, particularly in sacculated internal organs. Finally, the duration of certain key developmental stages has been elucidated, i.e., about 4 months for the development from an egg into an infectious larva and another 10 months for the further development into a mature adult, owing to a lifecycle duration of about 14 months under the given conditions.

Until today, only two cases of human pentastomiasis due to *A. agkistrodontis* have been described in China, one from Chinese Taipei (Taiwan; Republic of China) and another one from Hangzhou in Zhejiang province, People's Republic of China. The unique characteristics of the latter infection had been reported in 1996 [12]. Clinical features included long-term high fever, abdominal pain, diarrhea, mild anemia, hepatosplenomegaly, eosinophilia in both bone marrow and blood, and multiple polyps in the colon. The infective agent had been identified as *A. agkistrodontis* based on morphological and pathological observations as well as the reported dietary habits. Firstly, both eggs and juvenile parasites had been detected in the patient's feces, and the liver biopsy showed degeneration and necrosis of hepatocytes and an obvious infiltration with eosinophils. Second, recovered eggs were brown, oval and had a size of about 66–94 µm×33–57 µm. Third, the juvenile parasites measured about 21 mm×1.3 mm and had 7 abdominal annuli consisting of a cutin structure in bangle shape on their abdominal side. The parasite was enlarged in its anterior section, and had ventral hooks. Fourth, the patient had a history of drinking *A. acutus* gall and blood. The morphological features described in the reported case are consistent with the observations made during the present study. Additional findings of our study include the observation of anterior hooks, a cylinder-shaped larval body, an oval oral aperture with one pair of equal-sized hamuli on either sides, and 7–9 abdominal annuli with sucker-like sensory organs, providing additional indications for distinguishing *A. agkistrodontis* from *A. armillatus*.

The systematic classification of Pentastomida and the genetic relationship between different members of the group have been discussed extensively [22], [25], [26]. Over time, the group had been associated with a variety of metazoan phyla, including Arthropoda, Tardigrada, Annelida, Platyhelminthes and Nematoda. At present, their relationship with Arthropods/Crustacea is generally accepted, although the nature remains contentious [22], [27], [28]. Little taxonomic information on *A. agkistrodontis* has been published so far. In this study, a phylogenetic analysis based on the full 18S rRNA sequences showed that *A. agkistrodontis* is most closely related to *Raillietiella* spp., and a homology analysis of the Cox1 gene of *A. agkistrodontis* indicated that the sequence is most similar to the Cox1 gene of *A. armillatus*. Both species belong to the Pentastomida, and the phylogenetic analysis results confirmed that the pentastomids belong to the phylum Arthropoda [15].

We conclude that the full lifecycle of *A. agkistrodontis* can be established in the laboratory using a multi-host model. This will greatly facilitating future research into the biology, morphology and genetic makeup of Pentastomida, and provide material to work with for the development of diagnostic tools and the testing of drugs for treating this rare but arguably underestimated parasitic infection.

## Supporting Information

Alternative Language Abstract S1 Chinese translation of the abstract by QL.
(0.06 MB PDF)

## References

[1] Almeida WO, Vasconcellos A, Lopes SG, Freire EM (2007). Prevalence and intensity of pentastomid infection in two species of snakes from northeastern Brazil.. Braz J Biol.

[2] Almeida WO, Santana GG, Vieira WL, Wanderley IC, Freire EM (2008). Pentastomid, *Raillietiella mottae* Almeida, Freire and Lopes, 2008, infecting lizards in an area of caatinga, northeast, Brazil.. Braz J Biol.

[3] Lapage G (1959). Monnig's veterinary helminthology and entomology.

[4] Riley J, Henderson RJ (1999). Pentastomids and the tetrapod lung.. Parasitology.

[5] Almeida WO, Christoffersson ML (1999). A cladistic approach to relationships in Pentastomida.. J Parasitol.

[6] Fain A (1975). The Pentastomida parasitic in man.. Ann Soc Belge Med Trop.

[7] Riley J (1986). The biology of pentastomids.. Adv Parasitol.

[8] Anjos LA, Almeida WO, Vasconcellos A, Freire EM, Rocha CF (2007). The alien and native pentastomids fauna of an exotic lizard population from Brazilian Northeast.. Parasitol Res.

[9] Herzog U, Marty P, Zak F (1985). Pentastomiasis: case report of an acute abdominal emergency.. Acta Trop.

[10] Qiu MH, Jiang YY (2007). Distinguishing *Armillifer* and a correction on pathogen misidentification from a reported case.. Chin J Parasitol Parasit Dis.

[11] Qiu CP, Chang ZS, Tong XM, Zhang YN (2004). A boy infected with *Armillifer moniliformis*.. Chin J Parasitol Parasit Dis.

[12] Zhang Q, Wang B, Huang M (1996). *Armillifer agkistrodontis* disease, case report.. Chin Med J.

[13] Wang TP, Johansen MV, Zhang SQ, Wang FF, Wu WD (2005). Transmission of *Schistosoma japonicum* by humans and domestic animals in the Yangtze river valley, Anhui province, China.. Acta Trop.

[14] Giribet G, Ribera CA (2000). Review of arthropod phylogeny. New data based on ribosomal DNA sequences and direct character optimization.. Cladistics.

[15] Pinel C, Réjasse C, Picot S, Brenier-Pinchart MP, Grillot R (1999). *Blastocystis hominis*: réflexions épidemiologiques et cliniques à propos de plus de 3500 examens coprologiques.. Ann Biol Clin (Paris).

[16] Buckle AC, Riley J, Hill GF (1997). The in vitro development of the pentastomid *Porocephalus crotali* from the infective instar to the adult stage.. Parasitology.

[17] Stender-Seidel S, Thomas G, Bockeler W (2000). Investigation of different ontogenetic stages of *Raillietiella* sp. (Pentastomida: Cephalobaenida): suboral gland and frontal gland.. Parasitol Res.

[18] Lawn AM (1960). The use of potassium permanganate as an electron-dense stain for sections of tissue embedded in epoxy resin.. J Biophys Biochem Cytol.

[19] Johnson JE (1978). Transmission and scanning electron microscope preparations of the same cell culture.. Stain Technol.

[20] Antoniou M, Tselentis Y (1993). Studies on *Echinococcus granulosus* using the scanning electron microscope. I. Preparations of the parasite for infection of the final host.. Parasitol Res.

[21] Liu Q, Zhao JL, Zhou YQ, Liu EY, Yao BA (2005). Study on some molecular characterization of *Babesia orientalis*.. Vet Parasitol.

[22] Lavrov DV, Brown WM, Boore JL (2004). Phylogenetic position of the Pentastomida and (pan)crustacean relationships.. Proc Biol Sci.

[23] Esslinger JH (1962). Development of *Porocephalus crotali* (Humboldt, 1808) (Pentastomida) in experimental intermediate hosts.. J Parasitol.

[24] Buckle AC, Knox DP, Riley J (2002). Proteins and proteinases in the in vitro released products (IVRP) of the tissue-invasive and lung-dwelling larvae of the pentastomid *Porocephalus crotali*.. Parasitology.

[25] Waloszek D, Maas A (2005). The evolutionary history of crustacean segmentation: a fossil-based perspective.. Evol Dev.

[26] Zrzavy J (2001). The interrelationships of metazoan parasites: a review of phylum-and higher-level hypotheses from recent morphological and molecular phylogenetic analyses.. Folia Parasitol (Praha).

[27] Mallatt J, Giribet G (2006). Further use of nearly complete 28S and 18S rRNA genes to classify Ecdysozoa: 37 more arthropods and a kinorhynch.. Mol Phylogenet Evol.

[28] Almeida WO, Christoffersen ML, Amorim DS, Eloy ECC (2008). Morphological support for the phylogenetic positioning of Pentastomida and related fossils.. Biotemas.

