# Supplementary material for: Multi-host Model-Based Identification of Armillifer agkistrodontis (Pentastomida), a New Zoonotic Parasite from China
Source: PLoS Negl Trop Dis. 2010 Apr 6;4(4):e647. doi: 10.1371/journal.pntd.0000647 (PMC2850311; doi:10.1371/journal.pntd.0000647)
Supplement: Alternative Language Abstract S1 — Chinese translation of the abstract by QL. (0.06 MB PDF) [file pntd.0000647.s001.pdf]

摘要

背景：（Background）

舌形虫病是一类由人体充当中间宿主的罕见寄生虫病。尽管舌形虫其外形形似蠕虫，但根据其分类学地位舌形虫应归属于甲壳纲、节肢动物门，是雌雄异体的寄生虫。从其系统发生树来看，它在分类学上属独立的一个门。

方法与主要发现：（Methodology/Principal Findings）

在实验室用人工感染的方式建立了尖吻蝮蛇舌状虫的动物模型，在中间宿主小鼠、大鼠和终宿主蛇建立了生活史循环，并通过形态学和遗传学方法，阐明了尖吻蝮蛇舌状虫生物学特性。

用光镜和电子显微镜观察虫卵、幼虫、成虫各个不同阶段的形态特征。通过 18S rRNA 和 Cox1 基因核酸序列分析构建尖吻蝮蛇舌状虫的系统发生树。在小鼠体内，从虫卵感染发育到具有感染性若虫需要 4 个月，在终宿主蛇体内，从若虫感染发育为成虫需要 10 个月，因此，完成尖吻蝮舌状虫的完整生活史总共需要 14 个月。形态上，腕带蛇舌状虫（*A.armillatus*）和锯齿舌形虫（*Linguatula serrata*）的腹环数目有着明显的不同。

尖吻蝮蛇舌状虫（*A.agkistrodontis*）的基因克隆和同源性分析结果发现，尖吻蝮蛇舌状虫 CO1 基因的核酸序列与腕带蛇舌状虫（*A.armillatus*） CO1 基因核酸序列(AY456186)同源性最高，其氨基酸序列与腕带蛇舌状虫 CO1 基因氨基酸序列(YP025989)同源性最高；基于 18S rRNA 的进化树分析显示，尖吻蝮蛇舌状虫与蜥虎赖利舌虫（*Raillietiella* sp.）的遗传距离最近，它们同属于同一个分支，即五口虫纲。

结论：（Conclusion）

首次建立了可论证的罕见寄生虫尖吻蝮蛇舌状虫完整生活史的多宿主动物模型。多宿主模型的建立对阐明尖吻蝮蛇舌状虫属于甲壳纲、节肢动物门的分类学地位有着重要的意义。

关键词 （Keywords）：尖吻蝮蛇舌状虫；动物模型；生活史；博物学；人畜共患病；舌形虫；节肢动物门；中国
